# Bacterial Diversity of the Gastric Content of Preterm Infants during Their First Month of Life at the Hospital

**DOI:** 10.3389/fnut.2017.00012

**Published:** 2017-04-18

**Authors:** Laura Moles, Marta Gómez, Esther Jiménez, Gerardo Bustos, Javier de Andrés, Ana Melgar, Diana Escuder, Leónides Fernández, Rosa del Campo, Juan Miguel Rodríguez

**Affiliations:** ^1^Department of Nutrition, Food Science and Food Technology, Complutense University of Madrid, Madrid, Spain; ^2^ProbiSearch, S.L., Tres Cantos, Madrid, Spain; ^3^Servicio de Neonatología, Hospital Universitario 12 de Octubre, Madrid, Spain; ^4^Red de Salud Materno-Infantil y del Desarrollo (SAMID), Barakaldo, Spain; ^5^Instituto Ramón y Cajal de Investigaciones Sanitarias (IRYCIS), Madrid, Spain; ^6^Servicio de Microbiología, Hospital Universitario Ramón y Cajal, Madrid, Spain; ^7^Spanish Network for Research in Infectious Diseases (REIPI), Seville, Spain; ^8^Instituto de Salud Carlos III, Madrid, Spain

**Keywords:** preterm infants, stomach, gastric content, microbiome, microbiota

## Abstract

Studies focused on the stomach microbiota are relatively scarce, and most of them are focused on the adult population. The aim of this work is to describe the bacterial communities inhabiting the gastric content (GC) of preterm neonates. For that purpose, GC samples were collected weekly from a total of 13 preterm neonates during their first month of life within their hospital stay. Samples were analyzed by using both culture-dependent and -independent techniques. The former allowed the isolation of bacteria belonging mainly to the genera *Enterococcus, Staphylococcus, Streptococcus, Serratia, Klebsiella*, and *Escherichia*. The cultured dominant species in the GC samples during all the hospitalization period were *Enterococcus faecalis* and *Staphylococcus epidermidis*. Multilocus sequence typing (MLST) analysis revealed the presence of high-risk clonal complexes associated with the hospital environment, which may colonize enteral feeding tubes. Similarly, the 16S rRNA sequencing showed that *Streptococcus, Staphylococcus, Lactobacillus, Enterococcus, Corynebacterium*, and *Propionibacterium* were the dominant genera present at 75% of the gastric samples. However, the genera *Serratia, Klebsiella*, and *Streptococcus* were the most abundant. Own mother’s milk (OMM) and donor milk (DM) were collected after their pass through the external feeding tubes to assess their bacterial content. OMM and DM had a similar bacterial pattern to GC. Based on these data, the GC of preterm neonates is dominated by *Proteobacteria* and *Firmicutes* and harbors high-risk bacterial clones, which may colonize enteral feeding tubes, and therefore the feeds that pass through them.

## Introduction

The early colonization of the infant digestive tract is a complex process that has relevant consequences for health throughout the life span (1–7). Exposure to a myriad of microorganisms during the perinatal and neonatal periods is followed by a crucial sequence of active events leading to immune tolerance and homeostasis (8). The discrimination between commensal bacteria and invading pathogens is essential to avoid an inappropriate immune stimulation and/or host infection. The dysregulation of these tight interactions between host and microbiota can be responsible for important health disorders, including inflammation and sepsis.

Although full-term, vaginally delivered, and breast-fed infants are considered the ideal for the correct development of the gastrointestinal microbiota, in practice, there are many factors that may affect the acquisition, composition, and evolution of the infant gut microbiota, including gestational age, mode of delivery, diet, environment, or medical treatments (9–12). The initial colonization process is particularly challenging in preterm newborns, because of organ immaturity, higher rates of C-section deliveries, frequent use of antibiotics, and the stay at the hospital’s neonatal intensive care unit (NICU) (13). Under such conditions, it is not strange that preterm infants are frequently associated with an abnormal intestinal colonization pattern (14, 15), a fact that increases susceptibility to disease (16–18). Globally, the intestinal microbiota of preterm infants exhibits a significantly reduced bacterial diversity, an abundance of microorganisms usually related to hospital environments, and a reduced proportion of strict anaerobes with respect to facultative ones (19–23).

So far, gut has been the subject of most studies concerning gastrointestinal colonization, while studies dealing with the stomach’s microbiota are relatively scarce, and most of them are focused on the adult population with or without *Helicobacter* infection (24–31). In comparison to term neonates, the preterm’s stomach is characterized by the absence of periodical or rhythmic motility, a slow gastric emptying, and a relatively high gastric pH (5.5–7.0), facts that can be determinant in the establishment of the gastric microbiota (32–34). In addition, enteral feeding tubes (NEFTs) are often required to feed these babies but also contributed to the colonization by NICU-associated microorganisms (35, 36). As a consequence, the pass of any nutritional source [either own mother’s milk (OMM), donor milk (DM), or preterm formula] through the tubes may sweep along bacteria and have a strong impact on the infant intestinal colonization (36).

In this context, the objectives of this work were, first, to describe the bacterial communities inhabiting the gastric content (GC) of preterm neonates using culture-dependent techniques; such approach included the characterization of the bacterial lineages in the case of species frequently involved in preterm sepsis; and second, to apply the culture-independent techniques to describe the microbiome of a subset of samples.

## Materials and Methods

### Study Design

Thirteen preterm infants (≤32 weeks of gestation and/or ≤1,500 g of weight) of 31 recruited in a previous study (36) born at the Hospital Universitario 12 de Octubre of Madrid (Spain) were randomly selected for this study. Preterm infants with malformations, metabolic diseases, or severe conditions were excluded. Relevant demographic and clinical data such as antibiotherapy, enteral and parenteral nutrition, use of nasogastric tube, need of mechanical ventilation, hospital stay, birth weight, gestational age, gender, or delivery mode are described in Tables 1 and 2.

**Table 1:** **Demographic characteristics of the infants included in the study**.

Infant	Gestational age (weeks)	Gender	Birth weight (g)	Delivery mode	Sample collection (days of life)	
					Culture	16S rRNA
1	30	M	1,550	Cesarean section	0, 14, 21	0, 14
2	27	F	1,080	Cesarean section	0, 7, 14, 21, 28	0, 21
3	30	M	2,030	Cesarean section	0, 7, 14, 21	14
4	30	M	1,760	Vaginal	0, 7, 14, 21	14
5	32	F	1,310	Vaginal	0, 14, 21	14
6	26	F	920	Vaginal	7, 14, 21	21
7	29	F	1,040	Cesarean section	7, 21, 28	21
8	24	M	740	Vaginal	7, 14, 21	21
9	25	M	720	Vaginal	14, 21	21
10	28	M	1,100	Cesarean section	14, 21	21
11	31	F	1,430	Vaginal	0, 7, 14, 21	21
12	27	M	950	Cesarean section	7, 14, 21	28
13	24	M	870	Cesarean section	21	21
Mean (95% CI)	27.9 (26.3–29.5)		1,192 (950–1,435)			

**Table 2:** **Clinical characteristics of the infants included in the study**.

Infant	Antibiotherapy (days)	Parenteral nutrition (days)	Enteral feeding tube (days)	Mechanical ventilation (days)	Hospital stay (days)	*N* episodes of sepsis
1	3	5	38	0	42	–
2	9	3	48	0.5	60	–
3	3	0	26	2	27	–
4	0	0	26	0	27	–
5	3	0	21	0	28	–
6	33	8	97	26	102	2
7	7	5	45	0	47	–
8	27	13	112	35	116	1
9	38	10	144	140	144	1
10	7	14	62	10	73	–
11	3	4	35	0	37	–
12	3	8	62	0.04	70	–
13	29	12	90	37	102	–
Mean (95% CI)	12.7 (4.4–20.9)	6.3 (3.3–9.3)	62.0 (39.1–84.9)	0.5 (0–26)^a^	67.3 (44.2–90.4)	

*^a^Median (IQR)*.

Following the routine NICU feeding protocols, all infants were preferably fed with their OMM and, when this was not possible, with pasteurized human milk from the Milk Bank Unit DM. When the weight of the infants was ≥1,500 g and both types of human milk were unavailable, they received adapted preterm formula. In general, the feeding patterns of the recruited infants were very heterogeneous, a fact that prevented the formation of well-defined feeding groups.

Continuous nasogastric feeding was ordinarily administrated with a pump and intermittent feeding by gavage or pump. The syringe barrels were used as reservoirs that were connected through an external feeding tube (EFT). Feeding tubes were routinely replaced every 24 h, which means that different feed types could pass through the same tube during such a period.

None of the infants received antiacid treatment during their participation in the study.

### Samples Collection

Gastric content samples (~2 mL) were collected weekly by aspiration, using a sterile syringe, through the NEFT inserted into each baby’s stomach before new milk administration when intermittent feeding.

The feeding (OMM and/or DM) samples collected were the last fraction obtained after their passage through the EFT, immediately before entering the nasogastric tube at the connector, as previously reported (36).

All samples were collected 12 h after the replacement of the nasogastric feeding tubes and stored at −20°C until analysis.

### Culture Analysis

Adequate dilutions of GC samples were spread onto Man, Rogosa and Sharpe (MRS; Oxoid, Basingstoke, UK) and MRS supplemented with l-cysteine (0.5 g/L) (Sigma, St. Louis, MO, USA) (MRScys) for isolation of lactic acid bacteria, MacConkey (BioMérieux, Marcy l’Etoile, France) for isolation of *Enterobacteriaceae*, Baird Parker (BioMérieux) for isolation of staphylococci, Sabouraud Dextrose Chloramphenicol (SDC; BioMérieux) for isolation of yeasts, and Brain Heart Infusion (BHI; Oxoid), Wilkins-Chalgren (WC; Oxoid), and Columbia Nalidixic Acid Agar (BioMérieux) as general media for isolation of other bacterial groups. Plates were aerobically incubated at 37°C for up to 48 h, with the exception of SDC plates, which were incubated at 32°C for 96 h, and WC and MRScys plates, which were incubated (85% nitrogen, 10% hydrogen, and 5% carbon dioxide) in an anaerobic workstation (Mini-MACS Don Whitley Scientific Limited, Shipley, UK) at 37°C for 48 h.

### Bacterial Identification

Bacterial identification was performed as previously reported (36). The identifications were confirmed by matrix-assisted laser desorption/ionization time-of-flight mass spectrometry (Vitek MS, Biomerieux) at the facilities of ProbiSearch, SL. (Tres Cantos, Spain).

### Genetic Diversity of *Enterococcus faecalis, Staphylococcus aureus, Klebsiella pneumoniae*, and *Escherichia coli* Isolates

The genetic diversity of all the isolates belonging to the species *E. faecalis, S. aureus*, and *K. pneumoniae* was assessed by pulsed-field gel electrophoresis (PFGE) in a CHEF DR II apparatus (Bio-Rad, Birmingham, UK). To separate *Sma*I-digested fragments of enterococci and staphylococci, different protocols were applied (2–28 s for 24 h and 5–15 s for 10 h, and then 15–60 s for 13 h). The chromosomal DNA of *K. pneumoniae* isolates was digested with *Xba*I enzyme, and the electrophoresis conditions were 1–40 s for 20 h. The analysis of PFGE profiles was performed using the UPGMA method based on the Dice similarity by the Phoretix 5.0 software.

Multilocus sequence typing (MLST) schemes were applied for PFGE-unrelated strains of *E. faecalis* and *S. aureus*,^1^ *E. coli*,^2^ and *K. pneumoniae*.^3^

### DNA Extraction from the Gastric Samples

Gastric content samples were thawed at room temperature and centrifuged at 13,000 rpm and 4°C for 10 min. Then, the pellets were washed with TE buffer and centrifuged under the same conditions. DNA extraction protocol was carried out as previously described (23). To detect similarities and, more interesting, differences between GC and infant’s feeding, the bacterial composition of some OMM and DM samples was also assessed. DNA extraction of those samples was made following the same protocol.

### Next-Generation Sequencing Analysis

PCR amplifications were performed using primer 27F-DegL (5′-GTTYGATYMTGGCTCAG-3′) in combination with an equimolar mixture of two reverse primers, 338R-I (5′-GCWGCCTCCCGTAGGAGT-3′) and 338R-II (5′GCWGCCACCCGTAGGTGT-3′), generating ~345 bp amplicons from the V1 to V2 hypervariable regions of 16S rDNA genes. Barcodes used for Illumina sequencing were appended to 3′ and 5′ terminal ends of PCR amplicons to allow separation of forward and reverse sequences. Subsequently a bioanalyzer (2100 Bionalyzer, Agilent) was used to determine the concentration of every sample in the region of interest.

Barcoded PCR products from all samples were pooled at approximately equal molar DNA concentrations and run on a preparative agarose gel. The correct sized band was excised, and the DNA was purified. One aliquot of pooled, purified, barcoded DNA amplicons was sequenced on an Illumina MiSeq pair-end 2 bp × 250 bp protocol (Illumina Inc., San Diego, CA, USA) at the Unidad de Genómica of the Fundación Parque Cientifico de Madrid (Spain).

Raw sequences were processed according to their quality using the program TRIMOMMATIC by filtering those reads that showed a window of 50 bp with an average of quality values below 25. MOTHUR v 1.33.0 and UCHIME programs were used to eliminate chimeras and ambiguous bases. Resulting reads of quality controls were assembled and classified taxonomically by comparison with databases Greengenes, Ribosomal Database Project, and SILVA using a Bayesian classification method and a level of similarity of at least 97%.

### Statistical Analysis

The statistical analysis was performed using R 2.15.3 (R-project).^4^ When data were not normally distributed, median and interquartile ranges (Q1 and Q3) were calculated for all sampling times, and mean and 95% confidence interval (95% CI) were used for normal distributed data. The Kruskal–Wallis test for non-normal data or one-way ANOVA test when data were normally distributed were used to evaluate the differences between sampling times. In all cases, *P* values of <0.05 were considered to be significant.

## Results

### Characteristics of the Preterm Population

The 13 infants enrolled in this study had a mean gestational age of 28 weeks (ranging from 24 to 32 weeks) and a mean birth weight of 1,192 g (from 720 to 2,030 g) (Table 1). Approximately half of the infants (*n* = 7) were born by cesarean section (Table 1). All of them, except one, received antibacterial prophylaxis for, at least, the first 3 days of life and eight infants needed mechanical ventilation (Table 2). All infants were fed either with their OMM, DM, and/or preterm formula by nasogastric feeding tube for, at least, 21 days after delivery (mean of 62 days) (Table 2).

### Culture-Based Analysis of the GC Samples

A total of 38 GC samples collected during the first month of life were analyzed by culture-based methods: 6 samples from the first day of life (day 0), 8 from the first week of life (day 7), 11 from the second week of life (day 14), 11 from the third week of life, and 2 from the fourth week of life (day 28). Samples were very heterogeneous in texture and color, ranging from milky to mucous.

No microorganism could be isolated from 25% of the samples (particularly from those collected at day 0). When bacterial growth was detected, total bacterial counts oscillated between 4.20 and 5.95 log^10^ CFU/mL at day 0 and between 6.00 and 8.13 log^10^ CFU/mL at day 28 after birth in BHI media (Table S1 in Supplementary Material). The dominant bacterial genera detected were *Enterococcus, Staphylococcus*, and *Lactobacillus*, among Gram-positive bacteria, and *Klebsiella, Serratia*, and *Escherichia*, among Gram-negative ones. All of them showed a trend to increase, in both concentration and frequency, from birth onward, but only the genus *Enterococcus* showed a statistically significant increase during the study period (*P* = 0.027) (Table 3).

**Table 3:** **Microbial genera isolated from gastric content of preterm neonates**.

Genus	Day 0 (*n* = 6)		Day 7 (*n* = 8)		Day 14 (*n* = 11)		Day 21 (*n* = 11)		Day 28 (*n* = 2)		*P* value*
	*N* samples, *n* (%)	Bacterial counts, median (IQR)	*N* samples, *n* (%)	Bacterial counts, median (IQR)	*N* samples, *n* (%)	Bacterial counts, median (IQR)	*N* samples, *n* (%)	Bacterial counts, median (IQR)	*N* samples, n (%)	Bacterial counts, median (IQR)	
*Staphylococcus*	1 (17)	5.18	6 (75)	5.29 (1.27–5.99)	7 (64)	5.81 (3.35–6.23)	5 (45)	4.02 (2.30–5.85)	1 (50)	5.85	0.245
*Enterococcus*	0 (0)	–	2 (25)	4.66 (3.23–6.09)	5 (45)	4.19 (3.40–4.74)	8 (73)	6.25 (4.26–6.63)	2 (100)	3.58 (3.45–3.71)	0.027
*Streptococcus*	0 (0)	–	0 (0)	–	0 (0)	–	1 (9)	5.78	0 (0)	–	0.653
*Lactobacillus*	1 (17)	2.70	1 (13)	2.65	0 (0)	–	4 (36)	4.47 (3.43–5.02)	1 (50)	8.85	0.139
*Bifidobacterium*	0 (0)	–	0 (0)	–	1 (9)	2.00	0 (0)	–	0 (0)	–	0.653
*Klebsiella*	1 (17)	5.88	1 (13)	1.70	2 (18)	6.30 (6.17–6.42)	0 (0)	–	1 (50)	8.18	0.292
*Serratia*	1 (17)	2.70	2 (25)	6.05 (4.70–7.40)	1 (9)	6.88	2 (18)	6.14 (5.66–6.62)	1 (50)	9.06	0.575
*Escherichia*	0 (0)	–	0 (0)	–	2 (18)	3.33 (3.01–3.64)	1 (9)	5.22	1 (50)	2.78	0.269

*Friedman test.

**Figure 1:**
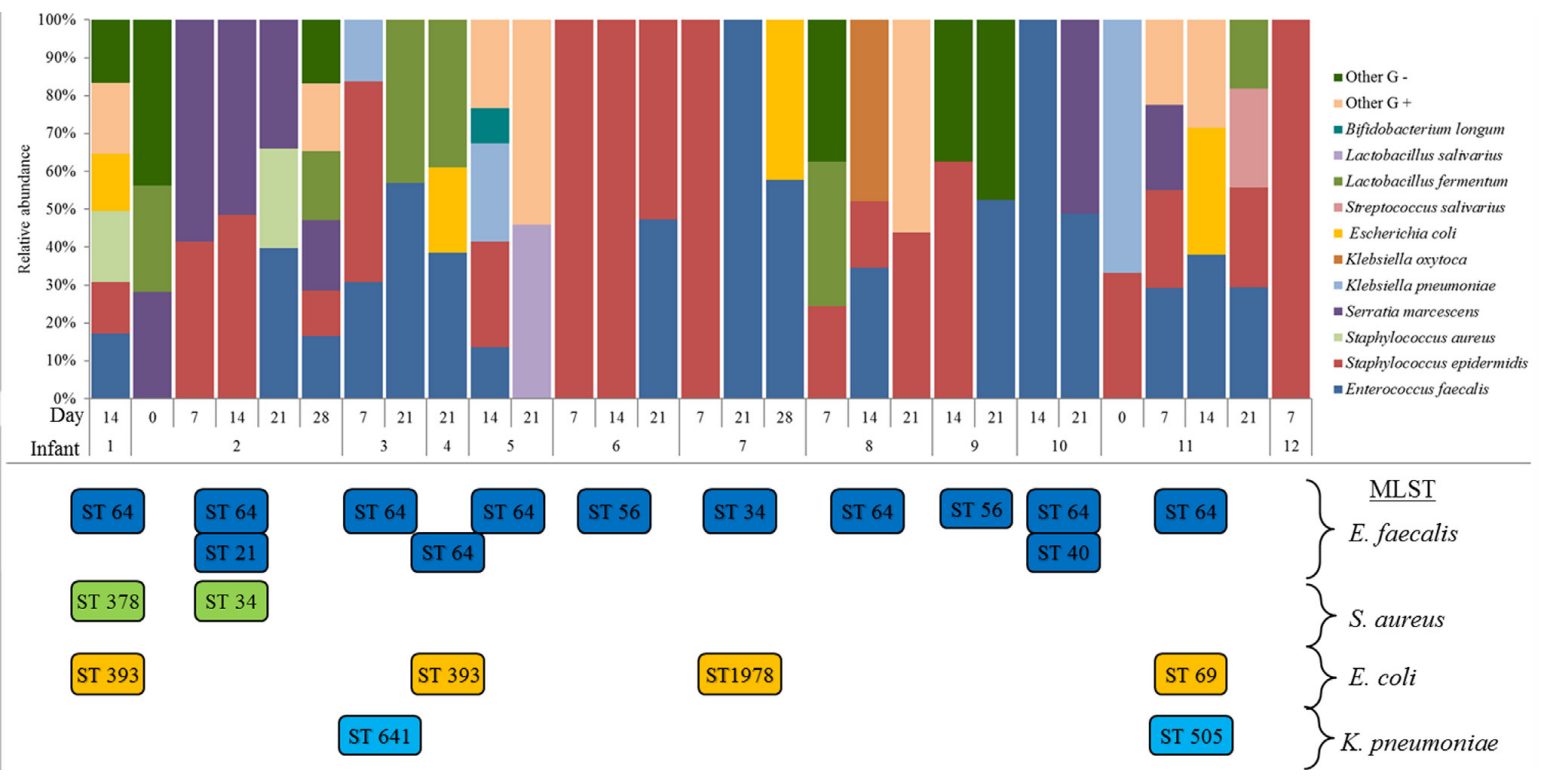
**Bacterial species isolated from the gastric content samples analyzed in this study**. ST clones of *Enterococcus faecalis, Staphylococcus aureus, Escherichia coli*, and *Klebsiella pneumoniae* detected by MLST genotyping in each infant and at each sampling time are shown below the graph.

A total of 241 isolates, belonging to 22 different species, were identified. The number of species per sample ranged from 1 to 17 species, and the bacterial profiles showed a high interindividual variability (Figure 1). Globally, the dominant species were *E. faecalis* and *Staphylococcus epidermidis*, followed by *Serratia marcescens, E. coli*, and *K. pneumoniae*.

### MLST Clones

MLST analysis revealed the presence of five different ST clones among the *E. faecalis* isolates. Most of the infants (67%) were colonized by the clone ST 64, while clone ST 56 was detected in two infants (6 and 9) and clones ST21, 34, and 40 only in one infant each (2, 7, and 10, respectively) (Figure 1). All these clones belonged to high-risk clonal complexes and are associated with hospital environments. In relation to *S. aureus*, ST378 and ST34 were isolated in GC of only two infants (1 and 2, respectively), but both clones are also associated with high-risk clonal complexes. The ST393, ST1978, and ST69 of *E. coli* were detected in gastric samples from four infants; two of them were colonized by the same clone (ST393). Finally, clones ST641 and ST505 of *K. pneumoniae* were detected in GC of two infants (3 and 11, respectively), and, again, both clones belonged to high-risk clonal complexes (Figure 1).

### Metagenomic Analysis of the GC and Milk Samples

**Figure 2:**
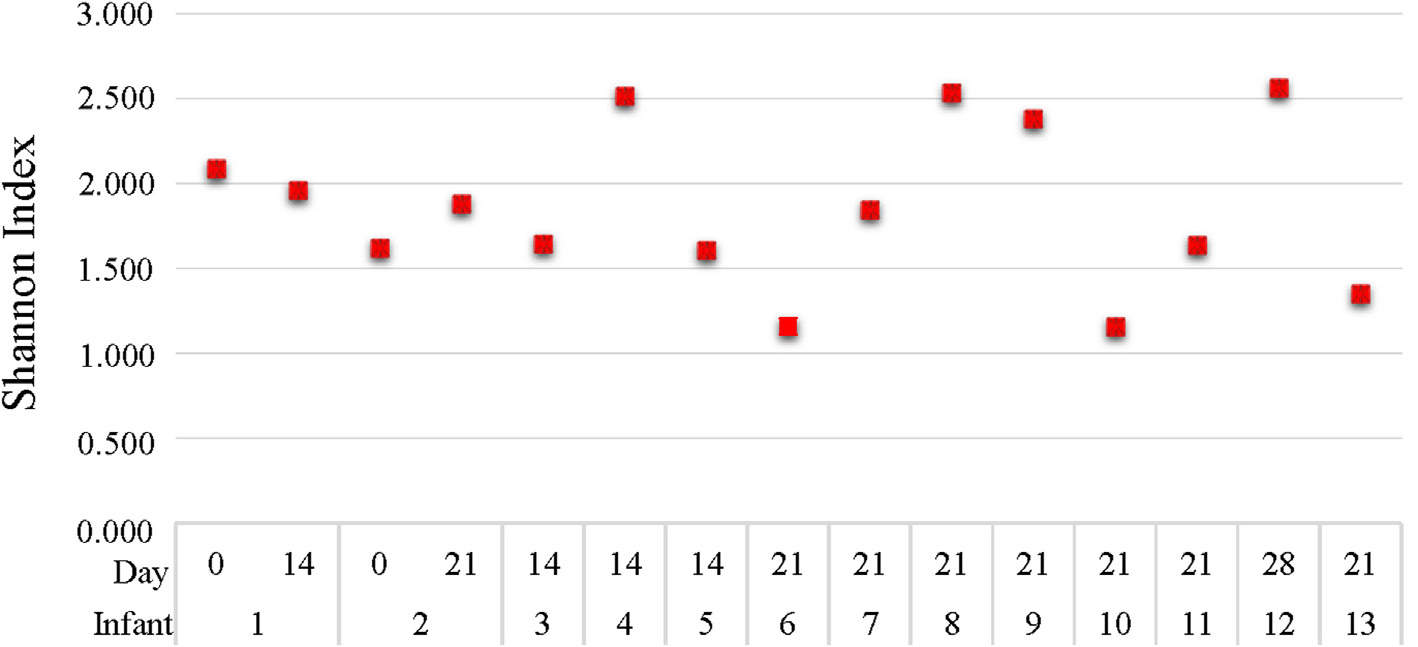
**Shannon diversity index of gastric content samples analyzed with 16S rRNA**.

DNA of good quality was obtained from 15 gastric samples of 13 infants. Two of them from the first week after birth (day 0), four from the second week of life (day 14), eight from the third week of life (day 21), and one from the fourth week of life (day 28). After processing the quality of the reads, a total of 3,613,468 sequences were analyzed that represented an average of 240,898 ± 32,493 sequences per sample. A high interindividual variability was detected among the samples. The Shannon Index ranged between 1.158 and 2.563 independently to the sample collection time with a mean of 1.863 ± 0.476 (Figure 2).

**Figure 3:**
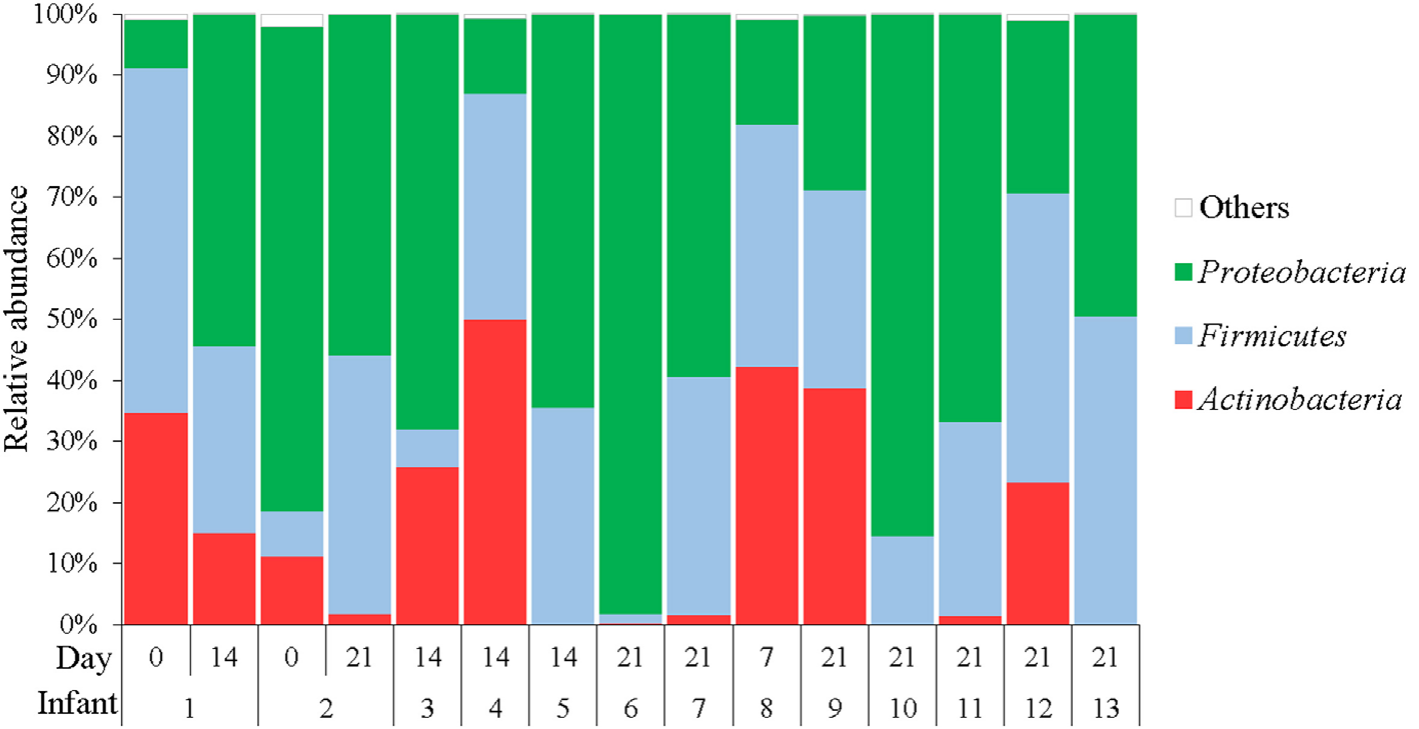
**Relative abundances of operational taxonomic units at phylum level accounting for >1% of the total bacterial community are shown for each sample**.

**Figure 4:**
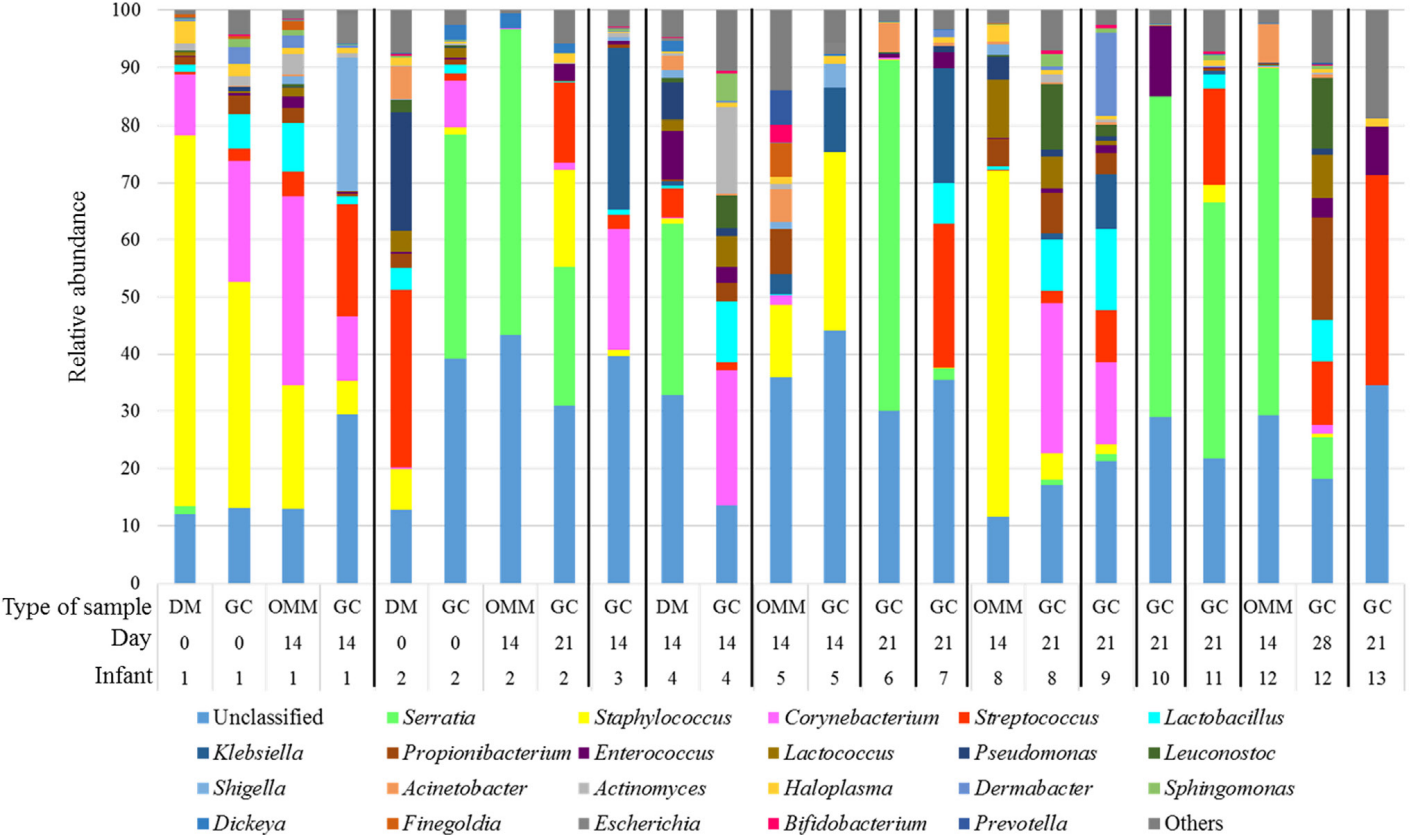
**Relative abundances of operational taxonomic units at genus level accounting for >1% of the total bacterial community are shown for gastric content (GC), own mother’s milk (OMM), and donor milk (DM) samples**.

Three phyla predominated in the analyzed samples, *Proteobacteria* (49.61% ± 14.69), *Firmicutes* (30.11% ± 8.86), and *Actinobacteria* (15.66% ± 9.44) (Figure 3). Other phyla were detected but with a relative abundance below 1%. Similarly, to the culture results, the most abundant genera observed in GC samples with 16S rRNA sequencing were *Corynebacterium, Streptococcus, Staphylococcus, Lactobacillus, Enterococcus*, and *Serratia*. However, 11 other genera represented that ≥1% of the communities were observed across samples (Figure 4). The genera *Serratia, Klebsiella*, and *Streptococcus* were most abundant in GC with a median relative abundance of 15.68, 9.59, and 9.07%, respectively (Table 4).

**Table 4:** **Relative abundance of bacterial genera represented ≥0.5% of the communities detected in gastric content (GC) and human milk samples**.

Phylum	Genus	GC, median (*n* = 15)	Own mother’s milk (OMM), median (*n* = 5)	Donor milk (DM), median (*n* = 3)	*P* value 1	*P* value 2
*Actinobacteria*	*Actinomyces*	0.58	2.16	0.36	0.197	0.735
	*Bifidobacterium*	0.46	1.62	0.20	0.558	0.127
	*Corynebacterium*	11.21	0.98	0.28	0.602	0.311
	*Dermabacter*	0.54	2.16	0.32	0.439	0.414
*Bacteroidetes*	*Prevotella*	0.28	5.87	0.13	–	0.221
	*Chryseobacterium*	–	0.22*	0.84*	–	–
*Firmicutes*	*Propionibacterium*	0.92	3.63	1.38	0.514	0.484
	*Enterococcus*	1.04	0.18	0.27	0.131	0.341
	*Gemella*	3.32*	–	0.09*		0.485**
	*Lactobacillus*	4.16	0.33	1.26	0.115	0.312
	*Lactococcus*	4.24*	5.79*	20.14*	0.515**	0.170**
	*Leuconostoc*	1.97	0.34	0.67	0.305	0.380
	*Staphylococcus*	2.41	17.12	7.11	0.833	0.112
	*Streptococcus*	9.07	0.10	5.10	0.122	0.062
	*Finegoldia*	0.42	1.43	0.65	0.655	–
	*Peptoniphilus*	0.41	1.96	0.11	1.000	–
*Proteobacteria*	*Agrobacterium*	0.06*	0.40*	1.12*	0.604**	0.180**
	*Dickeya*	0.14	1.39	0.98	0.296	0.117
	*Pseudomonas*	0.89	0.12	13.59	0.683	0.307
	*Sphingomonas*	0.71	0.12	0.27	0.229	0.157
	*Acinetobacter*	0.36	3.14	4.14	0.089	0.079
	*Klebsiella*	9.59	1.84	0.79	0.380	–
	*Leclercia*	1.39	0.05	0.22	0.134	0.739
	*Serratia*	15.68	56.86	15.68	0.133	–
	*Shigella*	0.57	1.34	0.79	0.655	0.770
*Tenericutes*	*Haloplasma*	0.78	1.29	1.44	0.086	–

*P value 1: Kruskal–Wallis was used to compare GC and OMM*.

*P value 2: Kruskal–Wallis was used to compare GC and DM*.

*Mean of the relative abundance.

**ANOVA test.

Gastric content samples were collected through the nasogastric tubes by syringe aspiration. Since this route was shared with OMM or DM samples, analyses of feeding samples by sequencing the 16S rRNA gene were also performed. Globally, a high degree of similarity between GC and milk samples was observed at the genus level, although the bacterial pattern of OMM samples were more similar to GC than DM (Figure 4). However, the statistical analysis did not show any significant difference (Table 4) between these three types of samples.

## Discussion

In this work, the microbiota and microbiome of the GC of a population of preterm infants during their first month of life at the NICU were studied. Traditionally, the human stomach was considered to be an inhospitable environment for microorganisms, with limited bacterial colonization and survival, because of the acidic conditions, fast peristalsis, and other antimicrobial factors. This led to the assumption that the human stomach did not harbor a complex microbiota. Initially, cultivation of gastric juice or mucosal biopsies identified several members of the *Firmicutes, Proteobacteria, Actinobacteria*, and *Fusobacteria* phyla in relatively low abundance (37). Bacterial viable counts in gastric luminal material usually ranges from non-detectable to 10^6^ CFU/mL (38), but the values may be strongly dependent on different factors, such as the part of the stomach, the actual gastric pH, diet, fastening time, or ethnicity (38, 39). The spectrum of values obtained in this work was also wide and within the range cited above.

The discovery of the genus *Helicobacter*, and the subsequent interest in the mechanisms by which these organisms adapt to the gastric environment, fueled research on the gastric microbiome (24–29, 31). The results of these studies have shown that the stomach’s microbiome is far more complex than initially expected and confirmed the dominance of this niche by members of the phyla *Proteobacteria, Firmicutes, Actinobacteria*, and *Fusobacteria*, in addition to some belonging to the phylum *Bacteroidetes*. *Streptococcus, Lactobacillus, Veillonella*, and *Prevotella* seem to be the dominant genera in healthy hosts, while *Helicobacter* was the most abundant in the human stomach of subjects who tested positive for this organism by using conventional clinical approaches. A vast number of microorganisms (>10^10^ CFU) may enter the human stomach every, day and as a consequence, a clear differentiation between truly resident from transient (swalled) microbial species is difficult. However, microbiome analysis has revealed that bacterial sequences in the stomach are not simply a random sampling of bacterial sequences from oral, upper respiratory tract, or esophageal communities, a fact suggesting that the presence of distinct bacterial communities adapted to the specific gastric environment (24, 40, 41).

In contrast to adults, there is an almost complete lack of data in relation to the microorganisms present in the GC of preterm neonates. Obviously, there are many environmental and medical differences among adults, term neonates, and preterms, and in addition, there are notable anatomical and physiological differences among the adult, the term neonate, and the preterm neonate stomachs; some of these factors may be determinant in the microbial composition of the preterm GC (32–34).

The culture approach used in this study allowed the isolation and characterization of a relatively ample collection of bacterial isolates belonging to hospital-associated species. Sepsis is one of the main causes of concern in the NICU, and therefore, hospital-adapted high-risk clones that exhibit antibiotic resistance and contain virulence factors are of the uppermost relevance (42, 43). The structure and main characteristics of the *E. faecalis, S. aureus, E. coli*, and *K. pneumoniae* populations were investigated, and, for these four species, high-risk clones linked to nosocomial infection were detected (e.g., *E. faecalis* ST64). In a previous study involving the same preterm population, a high proportion of antibiotic-resistant high-risk clones were detected in their fecal samples, suggesting a high degree of similarity between the fecal and the gastric microbiotas of preterm neonates (36). It has been reported that gastric hypochlorhydria in adults leads to an increased presence of intestinal bacteria in gastric samples (31).

Preterm infants are routinely tube-fed until they are physiologically ready for coordination of sucking, swallowing, and breathing, which often occurs at 33–36 weeks of postmenstrual age (44, 45). Therefore, any type of feed must be applied through the same feeding device as long as it is placed in a given neonate. Few studies have considered the role of neonatal NEFTs as a site of bacterial colonization and, consequently, as a source of bacteria for preterm infants and the influence of the feeding regime on the pattern of colonization of such devices. However, such studies have revealed the consistent presence of staphylococci (*S. epidermidis, S. aureus*), enterococci (*E. faecalis, Enterococcus faecium*) and *Enterobacteriaceae* (*K. pneumoniae, S. marcescens, Enterobacter cancerogenus, Enterobacter cloacae, E. coli*, and so on), including clones harboring antibiotic resistance genes, from the inner wall of most enteral feeding tubes analyzed so far (35, 36, 46). In a previous work (36), we detected the same bacterial genotypes of different genera in OMM, DM, and formula milk after their passage through the EFT and the fecal samples of different infants. SEM analysis of the internal surfaces of some sets of NEFTs revealed that complex microbial biofilms were formed when such devices were placed for at least more than 12 h. Aspiration of GC through the NEFTs was probably the source of bacteria proliferating in feeding systems and the source of bacteria that contaminated the milk samples. This would explain why, despite interindividual variability, the microbiome profiles of GC and milk samples were so similar in this study.

The 16S rRNA sequencing analysis revealed the dominance of two phyla (*Firmicutes* and *Proteobacteria*) in our gastric samples, which is in agreement with the results of a previous study (34). However, in the cited work, *Firmicutes* was the most abundant phylum, accounting for 50% of the reads, while *Proteobacteria* was the dominant one in our samples (33%). All the *Firmicutes* genera detected by Milisavljevic et al. (34) were also found in our samples, while there was no complete agreement regarding the bacterial genera belonging to the phylum *Proteobacteria*. In relation to the phylum *Actinobacteria, Corynebacterium* was the most abundant genus in both works. The presence of facultative anaerobic bacteria (streptococci, staphylococci, lactic acid bacteria, and enterobacteria, etc.) is a distinctive feature in the gastrointestinal tract of neonates, while strict anaerobes dominate in the adult gastrointestinal tract. This fact can explain why anaerobic bacteria are also dominant in the adult stomach (24–29, 31).

Globally, the results of this study show that GC of preterm neonates is dominated by Proteobacteria and Firmicutes and harbors high-risk bacterial clones, which may colonize enteral feeding tubes. Later, the preterm gastrointestinal tract is reinoculated with such bacteria when milk or preterm formula are administered through the same system. Therefore, future strategies to reduce bacterial contamination with high-risk clones of enteral feeding systems while preserving the potential transfer of beneficial bacteria should be devised. They may include a replacement of enteral feeding tubes as frequent as possible, the frame of a feasible NICU’s management, and/or the precoating of the internal surfaces of the tubes with probiotic bacteria specifically targeted for the inhibition of sepsis-related microorganisms.

## Ethics Statement

This study was carried out in accordance with the recommendations of the Ethical Committee of the Hospital Universitario 12 de Octubre with written informed consent from all subjects. All subjects gave written informed consent in accordance with the Declaration of Helsinki. The protocol was approved by the Ethical Committee of the Hospital Universitario 12 de Octubre.

## Author Contributions

LM, MG, EJ, GB, JA, AM, DE, LF, RC, and JR conceived and designed the experiments; LM, MG, EJ, JA, and RC performed the experiments; GB, AM, and DE recruited all the volunteers and collected the samples and the clinical data; LM, MG, EJ, JA, LF, and RC analyzed the data; LF, RC, and JR contributed with reagents/materials/analysis tools. All the authors wrote and read the paper.

## Conflict of Interest Statement

The authors declare that the research was conducted in the absence of any commercial or financial relationships that could be construed as a potential conflict of interest.

## Footnotes

^1^www.mlst.net.

^2^http://mlst.ucc.ie/mlst/dbs/Ecoli.

^3^http://www.pasteur.fr/recherche/genopole/PF8/mlst/Kpneumoniae.html.

^4^www.r-project.org.
