# Supplementary material for: Bacterial Diversity of the Gastric Content of Preterm Infants during Their First Month of Life at the Hospital
Source: Front Nutr. 2017 Apr 18;4:12. doi: 10.3389/fnut.2017.00012 (PMC5394887; doi:10.3389/fnut.2017.00012)
Supplement: Supplementary file 1 [file Table_1.DOCX]

**Additional Table 1. Bacterial counts in the samples of gastric content**

| Medium | Type of sample | No. Positive samples (%) | Mean  (95% CI) | Range of values | |
| --- | --- | --- | --- | --- | --- |
|  |  |  |  | Minimum | Maximum |
| BHI | 0 (n=6) | 2 (33%) | 5.08 (5.02; 5.13) | 4.20 | 5.95 |
|  | 7 (n=8) | 5 (63%) | 4.11 (1.27; 6.94) | 1.69 | 7.18 |
|  | 14 (n=11) | 8 (73%) | 5.93 (5.90; 5.96) | 3.70 | 7.81 |
|  | 21 (n=11) | 9 (82%) | 4.68 (4.64; 4.72) | 1.70 | 6.96 |
|  | 28 (n=2) | 2 (100%) | 7.07 (7.00; 7.13) | 6.00 | 8.13 |
| WC | 0 (n=6) | 2 (33%) | 4.56 (4.44; 4.67) | 2.70 | 6.41 |
|  | 7 (n=8) | 5 (63%) | 4.69 (1.61; 7.77) | 3.38 | 8.90 |
|  | 14 (n=11) | 8 (73%) | 6.18 (6.15; 6.21) | 3.85 | 7.79 |
|  | 21 (n=11) | 8 (73%) | 5.43 (5.38; 5.47) | 2.18 | 7.10 |
|  | 28 (n=2) | 2 (100%) | 7.72 (7.61; 7.82) | 6.00 | 9.43 |
| CNA | 0 (n=6) | 1 (17%) | 2.65 | 2.65 | 2.65 |
|  | 7 (n=8) | 5 (63%) | 5.12 (1.64; 6.64) | 2.65 | 6.41 |
|  | 14 (n=11) | 8 (73%) | 5.54 (5.51; 5.56) | 4.00 | 6.97 |
|  | 21 (n=11) | 9 (82%) | 4.78 (4.74; 4.83) | 2.00 | 7.01 |
|  | 28 (n=2) | 2 (100%) | 7.37 (7.28; 7.46) | 6.00 | 8.74 |
| BP | 0 (n=6) | 1 (17%) | 3.48 | 3.48 | 3.48 |
|  | 7 (n=8) | 4 (50%) | 3.98 (0.71; 7.26) | 5.18 | 6.47 |
|  | 14 (n=11) | 7 (64%) | 5.22 (5.18; 5.26) | 2.40 | 6.90 |
|  | 21 (n=11) | 4 (36%) | 4.52 (4.46; 4.59) | 1.70 | 6.23 |
|  | 28 (n=2) | 2 (100%) | 4.85 (4.78; 4.91) | 3.85 | 5.85 |
| MCK | 0 (n=6) | 1 (17%) | 5.33 | 5.33 | 5.33 |
|  | 7 (n=8) | 4 (50%) | 3.12 (0.53; 5.70) | 3.70 | 5.00 |
|  | 14 (n=11) | 8 (73%) | 5.20 (5.17; 5.24) | 2.98 | 6.88 |
|  | 21 (n=11) | 5 (45%) | 5.24 (5.20; 5.29) | 2.85 | 6.86 |
|  | 28 (n=2) | 2 (100%) | 5.95 (5.75; 6.14) | 2.78 | 9.11 |
| MRS | 0 (n=6) | 1 (17%) | 3.40 | 3.40 | 3.40 |
|  | 7 (n=8) | 4 (50%) | 3.00 (0.15; 5.85) | 2.93 | 6.84 |
|  | 14 (n=11) | 7 (64%) | 5.37 (5.33; 5.41) | 2.30 | 6.89 |
|  | 21 (n=11) | 7 (64%) | 5.59 (5.55; 5.63) | 2.00 | 6.82 |
|  | 28 (n=2) | 2 (100%) | 4.66 (4.58; 4.75) | 3.32 | 6.00 |
| MRScys | 0 (n=6) | 2 (25%) | 2.70 | 2.70 | 2.70 |
|  | 7 (n=8) | 2 (25%) | 1.47 (- 1.21; 4.15) | 2.60 | 6.23 |
|  | 14 (n=11) | 5 (45%) | 5.22 (5.18; 5.26) | 2.95 | 6.68 |
|  | 21 (n=11) | 6 (55%) | 4.94 (4.90; 4.98) | 2.00 | 6.69 |
|  | 28 (n=2) | 2 (100%) | 7.42 (7.33; 7.51) | 6.00 | 8.85 |
| SDC | 0 (n=6) | 0 (0%) | - | 0.00 | 0.00 |
|  | 7 (n=8) | 2 (25%) | 1.81 (- 1.41; 5.03) | 3.48 | 7.40 |
|  | 14 (n=11) | 3 (27%) | 5.19 (5.12; 5.27) | 2.81 | 6.76 |
|  | 21 (n=11) | 5 (45%) | 5.06 (5.00; 5.13) | 1.70 | 7.10 |
|  | 28 (n=2) | 1 (50%) | 5.28 | 5.28 | 5.28 |
